# Context and general practitioner decision-making - a scoping review of contextual influence on antibiotic prescribing

**DOI:** 10.1186/s12875-021-01574-x

**Published:** 2021-11-15

**Authors:** Resha Al-Azzawi, Peder A. Halvorsen, Torsten Risør

**Affiliations:** 1 General Practice Research Unit, Department of Community Medicine, UiT The Arctic University of Norway, PO Box 6050, Langnes, N-9037 Tromsø, Norway; 2 General Practice Research Unit, Department of Community Medicine, UiT The Arctic University of Norway, Tromsø, Norway; 3 Department of Public Health, Copenhagen University, Copenhagen, Denmark

**Keywords:** Decision-making, Context, Contextual factors, Contextual influence, Non-medical factors, General practice, Antibiotics, Prescribing

## Abstract

**Background:**

How contextual factors may influence GP decisions in real life practice is poorly understood. The authors have undertaken a scoping review of antibiotic prescribing in primary care, with a focus on the interaction between context and GP decision-making, and what it means for the decisions made.

**Method:**

The authors searched Medline, Embase and Cinahl databases for English language articles published between 1946 and 2019, focusing on general practitioner prescribing of antibiotics. Articles discussing decision-making, reasoning, judgement, or uncertainty in relation to antibiotic prescribing were assessed. As no universal definition of context has been agreed, any papers discussing terms synonymous with context were reviewed. Terms encountered included contextual factors, non-medical factors, and non-clinical factors.

**Results:**

Three hundred seventy-seven full text articles were assessed for eligibility, resulting in the inclusion of 47. This article documented the experiences of general practitioners from over 18 countries, collected in 47 papers, over the course of 3 decades.

Contextual factors fell under 7 themes that emerged in the process of analysis. These were space and place, time, stress and emotion, patient characteristics, therapeutic relationship, negotiating decisions and practice style, managing uncertainty, and clinical experience. Contextual presence was in every part of the consultation process, was vital to management, and often resulted in prescribing.

**Conclusion:**

Context is essential in real life decision-making, and yet it does not feature in current representations of clinical decision-making. With an incomplete picture of how doctors make decisions in real life practice, we risk missing important opportunities to improve decision-making, such as antibiotic prescribing.

**Supplementary Information:**

The online version contains supplementary material available at 10.1186/s12875-021-01574-x.

## Background

Decision-making is the backbone of everyday practice for general practitioners (GPs), and its research has thrived since the 1970’s [1]. Much of it focuses on the cognitive processes that occur, illustrating possible strategies used in the decision-making process [2–4]. Despite the abundance of research, how clinical decisions are made in real life practice remains elusive [1]. Diagnostic decisions and reasoning have been heavily favoured in published research, whilst what happens past the diagnosis stage remains unclear [5, 6]. Much of the research is based on imagined encounters, and focuses on how decisions *should* be made, rather than what actually occurs in practice [1]. It often presumes that when faced with similar cases doctors behave in a uniform manner [7], but variation is seen in all aspects of clinical practice including referrals [8], investigations [9], and prescribing [10]. There is intense debate surrounding this issue of variation between advocates of patient-centred care and evidence-based medicine. What promotes variation, when is it beneficial, and where should it be reduced to a minimum?

A growing body of evidence suggests that contextual factors may be related to this variation [10–12], although ‘context’ remains conceptually elusive. A pragmatic approach conceptualizes context as, “all that is expressed outside the boundaries of a patient’s skin that is relevant to planning the patient’s care” [13]. Examples include those specific to the patient such as gender or socioeconomic status, those specific to the GP such as experiences and tolerance of uncertainty, and those related the environment, such as culture and time constraints [13, 14]. This includes all so-called non-medical, and non-clinical factors and provides a pragmatic starting point for analysis.

From artwork that influences pain relief and stress levels, to lighting that reduces hospital stay length, contextual factors can improve patient care and reduce resource use [15–18]. Ignoring them can result in errors and adverse patient outcomes [13, 14, 19].

Despite the increase in studies, it remains unclear how context plays a part in decision-making in real situations. Studies have looked at influences on investigating, referring, overall management, and prescribing for numerous conditions. Studies have mixed GP, specialty physician and trainee decision-making [20]. When primary care has been the focus, nurse practitioners have been included [21].

We have undertaken a scoping review to gain better understanding of how GP decision-making and context interact, and what this means for the decisions made. We focus on decisions surrounding the prescribing of antibiotics, for a number of reasons. Increasing antibiotic resistance is a major challenge to global health. Serious infections attributed to resistant pathogens continue to rise in the UK, with a 32% increase noted between 2015 and 2019 [22], whilst the US reports over 35000 deaths due to resistance in 2019 [23]. 86% of antibiotic prescribing between 2015 to 2019 occurred in general practice [22], and despite efforts from the antibiotic stewardship movement, continues to be much higher than experts advocate [24]. Since the prescribing of antibiotics remains high despite all efforts to reduce it, we need to look beyond traditional measures. The decision to prescribe or not can be thought of as, on the surface, one of the “simpler” decisions one makes in general practice. It is also one of the more strictly “guidelined” and thus one would expect for context to play a less important role here.

This review aims to map the key contextual factors and their influence on GP decision-making in primary care by scoping those involved in antibiotic prescribing.

## Method

Medline, Embase, Cinahl databases were searched from January 1946 to October 2019. Reference searching of relevant papers and specific journal databases supplemented this. The search strategy was refined by RA and TR.

The protocol was created as per the Preferred Reporting Items for Systematic reviews and Meta-analysis Protocols Extension for Scoping Reviews (Prisma-ScR) [25] (Additional file 1).

### Inclusion criteria

We included English language papers, in primary care that discussed decision-making directly or indirectly related to GP prescribing of antibiotics*.* Any methodology was included, as were manuscripts that used any terms synonymous with context (full criteria and terms available in Additional file 1).

The original aim was to investigate the influence of context on all GP decision-making, but the terms ‘context’ and ‘contextual factors’ did not produce relevant articles. The search terms thus included “external factors”, “influencing factors” and “non-medical factors”. We also specified the factors relating to GP, patient, and environment. The large amount of data identified in this research made presentation of the findings long and difficult to convey in a meaningful manner. Brief analysis revealed an over-representation of papers focusing on prescribing antibiotics and so this became our focus. An additional search of the databases with the term antibiotic prescribing was performed (search strings provided in Additional file 1).

RA Screened the papers. Uncertainties were resolved with TR. RA charted the data into an Excel file based on Arksey and O’Malley’s charting form [26] including contextual factors described and documented influences on, initially all GP management, and then prescribing. RA and TR analysed factors in-depth looking for connections and whether effects were replicated, leading to grouping under seven themes. Conflicts on assignation of factors to themes were resolved via continued discussion.

Results are presented at the stage of the consultation in which the GP would experience them.

## Results

**Fig. 1:**
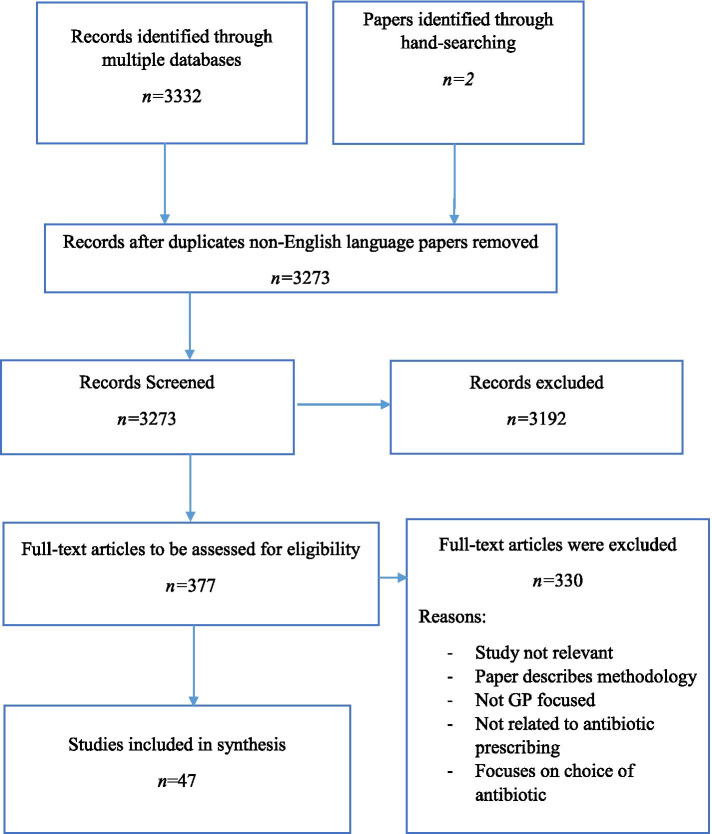
Flow chart detailing the eligibility process

The identification and selection process is illustrated in Fig. 1. We identified 3334 articles in total. Removal of duplicates and non-English language titles left 3273 papers for initial screening. Three thousand one hundred ninety-two records were excluded for not fulfilling all inclusion criteria. Three hundred seventy-seven full-text articles were assessed for eligibility. Forty-seven articles were included in the study.

## General trends

Contextual factors and their influence on prescribing fell under seven themes, summarised in Table 1 below.

**Table 1:** Contextual factors & influence on prescribing

Influence	Encourage prescribing	Reduce prescribing	Can encourage or reduce prescribing	Increase resource use/Affect decision-making/Influence health beliefs
Theme				
**Space & place**	Rural vs urban practice -rural practice can encourage prescribing		Culture	
**Patient characteristics**	Frequent Attenders Adverse Social Factors	Gender – reduced prescribing to women		Family & Friends Education Medical Knowledge
**Time, stress, & emotion**	Time Constraints Busy Hours Emotional Capacity of GP			Waiting Room Pressure
**Therapeutic relationship**	Fear for Dr-Patient Relationship	Knowledge of Patient		
**Negotiating decisions & practice style**	Patient Pressure Ease of Access to Antibiotics The Internet Patient Manner Rituals of Consulting Private Practice Patient Wishes & Expectations		Practice Style	Costs
**Managing uncertainty**	Uncertainty/Medico-legal Concerns Peers & Practice	Networks		
**Clinical experience**	Negative Experiences Habits Formed		Dynamic Behaviour	

Table 2 (Additional file 2) gathers all included research and tabulates the themes contained within each paper.

The findings reveal an increase in published papers over three decades, with the majority originating in the UK. Both quantitative and qualitative methodologies were well represented (43 and 51% respectively). Of qualitative research, only two papers observed consultations as part of their methodology.

The results are presented as a narrative using a fictional GP - Dr. Tea (DT) and her encounter with a patient - Joan Smith (JS). In weaving the findings into a recognizable scenario (based on the authors’ own experiences as GPs), not only are they more easily conveyed but it also proved invaluable in presenting the complexity of even a “simple” decision.

### Space and place

*DT is an experienced NHS GP working in a large city in the United Kingdom*

Prior to even the doctor and patient meeting, context can make its presence known. Societal attitudes towards healthcare varies between countries, but there has been a shift generally towards health anxiety and overconfidence in antibiotics, which promotes prescribing [27]. Greek society labelled low prescribers as less competent [28], whilst patients in Argentina, Russia and Lithuania were more likely to request and obtain antibiotics compared to those in Denmark and Sweden [29]. Having a robust professional medical culture, as seen in countries such as the UK, Denmark and Sweden encourages low prescribing rates [30].

DT practices in the city, but had she been more rural, the difficulty in accessing resources would encourage prescribing [27, 29, 31–33].

### Patient characteristics

*The first patient of the day, Joan Smith (JS), is a white, middle-aged, middle-class, retired high school teacher. She regularly discusses her health concerns with her husband, and close friends.*

Talking to family and friends influences health beliefs, which in turn can affect the consultation [34]. Patients’ educational attainment, and medical knowledge can also be crucial to decision-making [28, 35].

The evidence reveals that some patient characteristics encourage antibiotic use. These include being a frequent attender, and those with conditions such as alcoholism or homelessness [35, 36]. Gender also impacts on decisions made, with women being less likely to receive antibiotics as their symptoms are perceived to be less severe than men’s [34, 37, 38]. When a prescription is given, they tend to be for certain conditions, such as facial wounds [39]. Two papers found no evidence for the influence of gender [29, 33]

For other patient characteristics, the evidence is less clear. There is conflicting evidence on the effects age, socioeconomic status and patient ethnicity have on management decisions [29, 40–43].

### Time, stress, and emotion

*DT arrives late for work on a Friday morning. She is pre-occupied with thoughts of her ill child at home. Upon entering the already full waiting room, DT takes note of JS’s impatient facial expression.*

Unhappy patient facial expressions create “waiting room pressure”. This encourages shorter consultations, with less time for a thorough history and diagnosis, leading to increased resource use [44].

Time constraints brought about by increased workload also produces pressured consulting. This in turn increases prescribing, not only due to lack of time to negotiate management, but because the prescription can be used as a means of ending the consultation [27, 31, 37, 39, 40, 44–47]. There was conflicting evidence on whether increasing consulting times could change this [30, 48], and only one study refuted the findings [49]. More prescribing is also seen at busier hours, which are associated with more uncertainty such as the end of the day or out of hours [27, 30, 35, 40, 45, 50–53]. Not only do patients have higher expectations in these consultations, but the increased uncertainty also influences the interpretation of clinical signs, with infections seen more often at these times [50].

A doctor’s emotional capacity during decision-making can also have profound influences on actions. When tired or “having a bad day” they are less likely to take a history, examine or negotiate management [39, 40], which in turn encourages prescribing [27, 40, 51].^.^

### Therapeutic relationship

*With her complex medical history, and repeat attendances, JS is well known to DT.*

The doctor-patient relationship is central to management decisions. A good knowledge of the patient reduces prescribing [30, 33, 52, 54, 55]. Doctors felt that this was supported by taking account of the patient in front of them as opposed to just applying guidelines to all [30]. Fear for this relationship can encourage the use of the prescription as a means of improving or maintaining it [27, 28, 36, 54, 56]. Some GPs feel that if they did not prescribe, the patient would simply see another doctor that would [32, 54, 57].

### Negotiating decisions and practice style

*DT listens to JS explain how she has had a niggling cough for over a week. Her husband has researched her symptoms on the internet and has nagged her to get it checked. Following assessment, DT feels that there is probably no need for antibiotics. JS expresses concern that her husband will not be happy for her to come back empty handed, especially when it is nearly the weekend. She also mentions that DT’s colleague, Dr. Mann, who she sees on occasion always gives her antibiotics when she has a cough.*

The internet provides ever-increasing avenues of information for patients, reliable or otherwise. Although this has empowered patients, it also generates worries that can pressure DT to lower her prescribing threshold [27, 29, 32]. This patient pressure is a strong influence for prescribing [27, 28, 53], and is interwoven into practically all decision-making [46]. One study quoted a prescribing rate of 83% when pressured by patients [58]. Connected to this, is the “chagrin factor” where GPs prescribe when pressured, as it causes more chagrin not to [59]. Combine this with easy access to over-the-counter antibiotics, or an aggressive patient manner, and refusing a prescription is even harder [28, 29, 39, 35, 60]. GPs in the private sector feel even more pressure to prescribe to keep patients happy [33, 44, 59].

One outlier study found no influence of patient pressure on prescribing rates [49].

Patient wishes and expectations, whether directly expressed or simply assumed also increase antibiotic use [47, 55–57, 59–64]. This prescribing has no influence on the perceived probability of an infection [61]. In one vignette-based study, GPs considered patient demands in 50% of prescribing decisions [65]. Certain consultations carry with them different patient expectations and are an important part of management decisions [29]. In out of hours or private work, particularly, there is a greater expectation for a prescription as an exchange for the effort of attending, which in turn pressures the GP into feeling that they should meet expectations [66].

Style of practice, and the opinions held on the role of a GP also affects management decisions. Those with a service minded mentality, and views of prescribing as an essential tool are more likely to prescribe in comparison to those with a more biopsychosocial style [27, 28, 60, 67].

The act of prescribing can signify not just infection. It can signal that the patient’s symptoms are being taken seriously [32]. It can end the consultation or prevent re-attendance. It can also be used as a bargaining chip [40, 51, 56]. The use of prescriptions in this manner make it difficult to refuse a script, no matter how inappropriate [51], and so raises prescribing rates [27, 29, 67]. Some diagnoses become so closely bound with antibiotics that a prescription is seen as part of the ritual of consulting for this type of illness [32, 57].

Costs to both the patient and the system are also an important aspect of managing decisions [28, 40, 68–70].

### Managing uncertainty

*DT agrees to give a delayed prescription, with instruction on when to use it.*

JS’s obvious discontent with the prospect of leaving the consultation empty handed can raise feelings of uncertainty and medico-legal concerns in DT. These fears promote prescribing [27, 45, 46, 39, 50, 36, 54, 67, 71]. Maintaining a restrictive attitude to prescribing can be difficult to maintain over the course of the day [39].

JS mentions DT’s colleague and his usual practice. Relationships with her peers can play a big part in decisions. From fear of appearing incompetent in comparison to, or in front of colleagues [28], to difficulty deviating from usual peer practices [46, 40, 56]. Good networks with not only her peers, but also her allied practice staff can positively influence prescribing [27, 28, 30, 37, 72].

### Clinical experience

*As the door closes behind JS, DT reflects on what happened. Why did she write a prescription? Clearly, something must have influenced her… But then the image of the full waiting room enters her mind, and she shakes off the thought and hurries onto the rest of her day.*

DT has been practicing for some time. Prescribing behavior can show dynamic changes, with rates waxing and waning over the years, and these decisions are guided by not only policy but also by pressures of every day practice [36]. These context-guided decisions are only used in specific patient cases assigned by the GP, and in this way, context could influence DT to switch from behaving as a low prescriber to a high one. Others have shown both a softening of stance, and less prescribing with experience [54, 67].

Experiences during her many years of practice can also impact on decisions [36, 65, 68, 70]. Dramatic or negative events, such as missed diagnoses, adverse drug reactions, unexpected deaths or even the experience of taking antibiotics themselves increased prescribing [47, 51, 36, 72]. Habits can also play a part, with evidence to show that patients are prescribed antibiotics because the GP normally prescribes for a particular disease, not because of clinical factors [73].

## Discussion

This review displays just how vital context is to patient management, and so should be of importance to not only the academic community and antibiotic stewardship movement, but also those busy GPs like Dr. Tea, and all involved in molding the next generation of doctors.

Contextual presence gave a supposedly “simple” decision – to prescribe antibiotics or not - a level of complexity not easily recognizable before. It was prominent at every step of the consultation, even before doctor and patient come face-to-face.

Being dynamic in nature, context, somewhat paradoxically, both gives direction and focus for consultations, but also ensures that no two consultations are the same. In many papers, context in comparison to signs, symptoms and evidence, was seen as a hindrance to good decision-making. Nevertheless, the evidence here suggests that the focus on symptoms and evidence may be too narrow since context and experience are crucial in guiding real-life decisions too [36].

Research on this topic has shown an increase over the three decades reflecting recognition of its importance, but numbers are small when compared to the rise of published scientific literature in general [74].

One would expect that context would make more of an impact on the most important parts of the consultation – particularly that of history-taking and examination and yet the data show very little there. Arguably, this is more likely due to very few studies observing the history taking and examination process, rather than an actual lack of importance.

Our work has given a comprehensive overview of the evidence on context and prescribing antibiotics, as well as highlighted research gaps and methodological concerns.

### Strengths and weaknesses

This is the first paper to our knowledge to present the level of complexity inherent in even “simple” decisions in such a way. It also highlights that they may not be simple at all. The inclusion of both quantitative and qualitative works have allowed for richness of data. Detailing the findings from many countries illustrates not only the ubiquity of context but gives a broader picture of the connections between decision-making and both societal and professional norms.

The research is English language based and thus presents a potential publication bias, and the possibility of discounting important findings based on language.

Another limitation concerns unaddressed factors, such as those relating to seasonal variation of prescribing, the influence of antibiotic stewardship systems and local resistance data. This might represent missed research due to lack of consensus on defining “context”, as opposed to lack of research. Papers examining, for instance, the influence of consultation length on prescribing might not consider it a contextual factor. Directly related, is that the study by design, when using terms such as “non-medical factors” is likely to retrieve papers that consider context as a problem in decision-making.

### Comparison with existing literature

The absence of a unifying definition of context creates challenges to furthering our understanding of decision-making. Geertz’s idea of ‘culture’ may better describe what we term ‘context’. He describes man as being “suspended in webs of significance he himself has spun”, and it is these webs that represent culture [75]. Analysis of these webs is more about discovering meaning rather than scientific laws. If we understand context to be these webs of significance that we spin in our practice, then the study of it in decision-making could less focus on measuring impact, as opposed to the meaning in the decision-making process.

Theories of medical decision-making make no mention of context in the form presented here. These include both normative theories, that suggest the same processes occur in all decisions, and descriptive theories that aim to explain what actually occurs. Among the exceptions is the “choice architecture” theory of Thaler and colleagues [76]. They use this term “choice architecture” to, in essence, describe context. They discuss how decisions are not made in a vacuum, and that the environment, with features both “noticed and unnoticed” can have great influence on decision-making. They go further by suggesting that one can improve decision-making by altering context. This resonates with the findings here.

The argument for the deep-rooted, intrinsic nature of context in primary care decision-making has been emphasized by several authors including McWhinney, and Malterud [77–79]. Context could help understand the variation found in decision-making. Doctors prescribe for many reasons other than a diagnosis, and prescriptions do not necessarily reflect the perceived probability of disease [61]. It was a complex matrix of ever-changing factors that informed management, not just clinical judgements and guidelines, which is consistent with Situativity Theory and the findings of Durning and colleagues [6, 12, 80].

The focus on diagnosis and guidelines prevents the contextualization of clinical decision-making in a way that allows context to be a legitimate part of the process. Contextual presence is viewed as a negative influence outside of the traditional clinical domain, exerting its power as an external force on the GP [35, 60]. It has been described as “something other than the essential content (that) is driving the physician’s clinical reasoning” [81]. For example, several studies have focused on guidelines adherence as outcomes. Clinical trial data, which purposefully removes context, forms the basis for guidelines [13]. If the starting point is to highlight why GPs do not follow guidelines, or why there is “non-pharmacological” prescribing, then any variation may be labelled unwarranted.

The evidence reveals that contextual influences are neither non-clinical nor non-essential. They are bound to the encounter and are, in essence, as clinical as any sign or symptom as they hold influence over the actions of the doctor. What is deemed “clinical” in most research is actually only a small part of what is considered “clinical” from a practical perspective. In cases where patients present with symptoms suggestive of serious disease, there is less variation in management and less information makes for better outcomes [82]. One could make the conclusion that in these cases context is not as important or relevant for the practical management of care. However, if the evidence shows that context is present in all aspects of the encounter, is it really so easy to ignore in serious scenarios, or could it be that it simply directs management towards protocol, whatever that is depending on context? One example being acute MI in rural areas vs urban areas, where the management is still to “unblock” the blockage by the means available – PCI in urban and thrombolysis in rural.

### Implications for clinical practice

The promotion of context in clinical encounters, and its inclusion in education, clinical practice, and policymaking, has great potential to improve patient care.

We as clinicians endeavour to make decisions that are objective, considering only the clinically relevant. However, if we examine Dr. Tea’s consultation we can see how unconsciously our everyday decisions are more organic and embedded. When tired, when facing the prospect of a full waiting room or the beginning of the weekend these all can alter the outcome of our consultations. Reflective practice and dialogue between peers about real life outcomes, and the ways in which context may have played a part in these would help ensure best quality care.

### Implications for policy makers

This research presents the possibility of truly making an impact on antibiotic prescribing by focusing on researching those influences that can be altered. For example, can prescribing practices be improved by tackling society’s ideas around antibiotics and health? Can they be changed by increasing consultation times, particularly at traditionally busy hours? Norwegian data shows a reduction in prescribing rates under the context of clinical practice during a pandemic, revealing the very real impact context can have [83].

### Implications for clinical reasoning research

The literature presented heterogenous conceptualisations of context and methods of identifying it. There were numerous terms used, with few expanding on what they meant by them. When papers have attempted to define context, they focused on the patient, without specifically mentioning the physician [13, 14, 84]. With no universally accepted definition of “context”, one cannot be sure that what is being investigating is the same in each paper.

Methodologies ranged from extrapolating from notes, from prescriptions, from interviews, questionnaires and focus groups. Only two papers used actual observed encounters as a basis for their study. When looking at notes, or prescriptions one is looking back at the encounter from the endpoint. The consultation with Dr. Tea shows how this might be problematic from a quality point of view – each part of the consultation brought with it a layer of factors that could alter the direction of management. Future research would benefit from observation of real encounters to obtain a more accurate picture of clinical practice. This paper touches on only a small part of the decisions GPs, and doctors in general make [85]. Further research is needed to explore how context plays a part in others.

If our understanding of decision-making in primary care presumes that diagnosis is the goal of all consultations then it is, at best, partial. These encounters have low disease prevalence, and decisions often occur before a formal diagnosis [6].

Studying management reasoning, where contextual factors are included, could pave the way forward [5, 6].

### Implications for medical education

Inclusion of context as an integral part of diagnosis and management would present the reality of practice and help smooth the transition from medical student to practicing clinician.

## Conclusions

Context is essential in real life decision-making, as exemplified by antibiotic prescribing, and yet it does not feature in current representations of decision-making/clinical reasoning research. With an incomplete picture of how doctors make decisions in real life practice, we risk missing opportunities to improve decision-making, such as antibiotic prescribing.

## Supplementary Information

**Additional file 1.**

**Additional file 2.**

## Data Availability

All data generated or analysed during this study are included in this published article. Study protocol is attached as an Additional file.

## Abbreviations

GPs: General practitioners
