# Supplementary material for: Context and general practitioner decision-making - a scoping review of contextual influence on antibiotic prescribing
Source: BMC Fam Pract. 2021 Nov 15;22:225. doi: 10.1186/s12875-021-01574-x (PMC8591810; doi:10.1186/s12875-021-01574-x)
Supplement: Supplementary file 1 — Additional file 1. [file 12875_2021_1574_MOESM1_ESM.docx]

Review Protocol for Contextual Factors and GP Decision-Making:

Background:

Decision-making is the backbone of everyday practice for general practitioners (GPs), and its research has thrived since the 1970’s. Much of it has focused on the cognitive processes that occur, illustrating possible strategies used in the decision-making process. Despite the abundance of research, how clinical decisions are made in real life practice remains elusive.

A growing body of evidence suggests that contextual factors may be related to the variation seen, although ‘context’ remains conceptually elusive. Despite the increase in studies, it remains unclear how context plays a part in decision-making in real situations.

We have undertaken a scoping review to gain better understanding of how GP decision-making and context interact, and what this means for the decisions made. After reviewing the data, we have chosen to focus this on decisions surrounding the prescribing of antibiotics as it presents a major challenge to global health, there is an abundance of research and it represents a “simple” decision.

Objectives:

The main research questions is “What is known about how external factors/non-medical factors/contextual influence GP decision-making?”

Update - As data on all decisions made was too vast for one paper, objectives changed to focus on external factors/non-medical factors/contextual influence on GP prescribing of antibiotics. As such the research question has changed to “what is known about how contextual factors influence GP antibiotic prescribing?”

Methods:

A scoping review agreed as the most appropriate methodology as a starting point.

RA and TR – discussed terms to be entered in search. Table 1 created, used as foundation for search terms.

Inclusion criteria:

- Published peer-reviewed research
- In Medline, Embase and Cinahl databases
- English language
- Primary care setting with a focus on GPs
- Related to prescribing of antibiotics

Exclusion criteria:

- Non-English language text
- Primary care setting with focus on all healthcare staff prescribing
- Studies where full-text article could not be obtained

All eligible articles will be added to Endnote software, with removal of duplicates.

RA will conduct full-text screening of selected studies, with discussion with TR should uncertainty arise.

Charting the data:

The selection and data-charting process will follow the Preferred Reporting Items for Systematic Reviews and Meta-Analyses Extension for Scoping Reviews (PRISMA-ScR) recommendations. Data charting will be performed by RA, with discussion with TR. The following will be charted into Excel software:

- Title of study

- Authors

- Year of study

- Location of study

- Aims

- Study population

- Methodology

- Data collection methods

- Study findings including:

- Contextual factors identified
- Influence on decision-making/antibiotic prescribing

Analysis and reporting of results:

RA and TR will analyse data and aim to connect the factors under common themes. This will be entered into Excel software. Results will be reported as they occur in the consultation process to illustrate the flow of decision-making.

Discussion:

The planned scoping review aims to review contextual factors influencing GP decision-making. This is now updated to reviewing contextual factors influencing GP antibiotic prescribing. This review will be of interest to academic GPs with an interest in decision-making, those in the antibiotic stewardship movement, the busy working clinician and those that teach.

Limitations discussed include those around language, the lack of consensus around defining or recognising contextual factors, and the potential focus on negative findings.

# red= mesh term

| GP | Uncertainty | Decision making | External Factors |
| --- | --- | --- | --- |
|  |  |  |  |
| General practitioner* | Uncertainty | Decision*  Decision making –expand only specific ones  Decision making, organizational | Non-medical factors  Professional-family relation  Professional-patient relation  Organizational culture |
|  |  |  |  |
| Family practitioner* | Anxiety – don’t explode | Physicians’ practice/patterns  Institutional practice – includes professional practice | Influencing factors |
| Family practice | Risk | Clinical decision-making | Physician-patient relations |
| General practice | Risk-taking – don’t explode | Cognition – includes imagination, and intuition. | Regional factors |
| Physicians, family | Ambiguity | Diagnostic tests | Interprofessional relations |
|  |  |  |  |
| ~~Primary care~~ | Medically unexplained symptoms | Referral & consultation | Personality |
| General practice physician* | Curiosity (exploratory behavior) | Unnecessary procedures (under health service misuse) | Age factors |
| ~~Primary care physician*~~  family doctor  primary health care  physician, primary care | Wonder/wondering  Choice  options | Practice patterns, physicians’ | Sex factors |
|  | Un-explained compaints | Medical Overuse – included in health service misuse | Time factors |
|  | Diagnostic uncertainty  somatoform disorder* | Attitude of health personnel  Diagnosis  Diagnostic services/utilization  Medical overuse/prevention & control  ?decision theory  Diagnostic approach  Diagnostic technique & procedures – includes clinical lab technique, imaging and diagnostic tests, routine  Health services accessibility  Risk management  Risk-reduction behaviour  Reasoning  Thinking – includes creativity, decision making and clinical judgement.  Delivery of health care – includes practice patterns,physicians, professional practice gaps and telemedicine | Female  Male  Social class  Ethnic groups  ~~Family~~  ~~Relations~~  Context  Culture - ?included in institutional practice  complexity |
|  |  |  |  |
|  |  |  |  |
|  |  |  |  |

*Table 1 – Terms For Use in Search Strings*

Search strings with best results for GP decision-making:

MEDLINE:

All GP decisions:

1. exp Physicians, Family/ or exp Family Practice/ or exp General Practitioners/

2. exp General Practice/ed, st, sn [Education, Standards, Statistics & Numerical Data]

3. exp Physicians/px, sn, ut [Psychology, Statistics & Numerical Data, Utilization]

4. exp Primary Health Care/sn, ut [Statistics & Numerical Data, Utilization]

5. 1 or 2 or 3 or 4

6. exp Uncertainty/

7. exp Somatoform Disorders/di, px [Diagnosis, Psychology]

8. 6 or 7

9. exp Decision Making/

10. exp Clinical Laboratory Techniques/px, sn, ut [Psychology, Statistics & Numerical Data, Utilization]

11. exp Diagnosis/

12. exp "Attitude of Health Personnel"/ or exp Practice Patterns, Physicians'/

13. (referral and consultation).mp. [mp=title, abstract, original title, name of substance word, subject heading word, keyword heading word, protocol supplementary concept word, rare disease supplementary concept word, unique identifier, synonyms]

14. exp Unnecessary Procedures/px, sn, td, ut [Psychology, Statistics & Numerical Data, Trends, Utilization]

15. exp Decision Theory/

16. exp Medical Overuse/pc, sn, td [Prevention & Control, Statistics & Numerical Data, Trends]

17. 9 or 10 or 11 or 12 or 13 or 14 or 15 or 16

18. influencing factors.mp.

19. non-medical factors.mp.

20. physician-patient relations.mp. or exp Physician-Patient Relations/

21. exp Personality/ or personality.mp.

22. interprofessional relations.mp. or exp Interprofessional Relations/

23. age factors.mp. or exp Age Factors/

24. sex factors.mp. or exp Sex Factors/

25. time factors.mp. or exp Time Factors/

26. ethnic groups.mp. or exp Ethnic Groups/

27. Female/

28. Male/

29. external factors.mp.

30. 18 or 19 or 20 or 21 or 22 or 23 or 24 or 25 or 26 or 27 or 28 or 29

31. 5 and 8 and 17 and 3

Update:

Antibiotic prescribing:


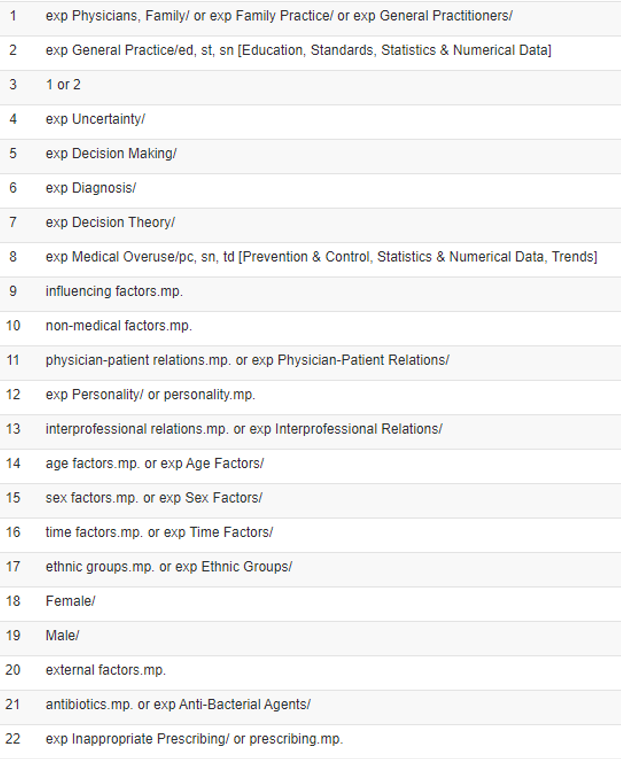


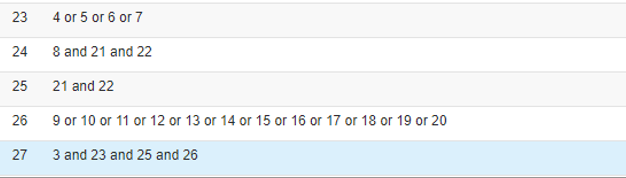


EMBASE:

All GP decisions:

1. exp general practitioner/

2. exp general practice/

3. exp clinical practice/

4. exp patient referral/

5. exp primary health care/

6. uncertainty/

7. risk/

8. exp diagnostic reasoning/

9. exp clinical decision making/ or exp medical decision making/ or decision making/

10. diagnostic test/

11. exp health personnel attitude/ or exp physician attitude/

12. exp problem solving/

13. 1 or 2 or 5

14. 6 or 7

15. 3 or 4 or 8 or 9 or 10 or 11 or 12

16. 13 and 14 and 15

Update:

Antibiotic prescribing:


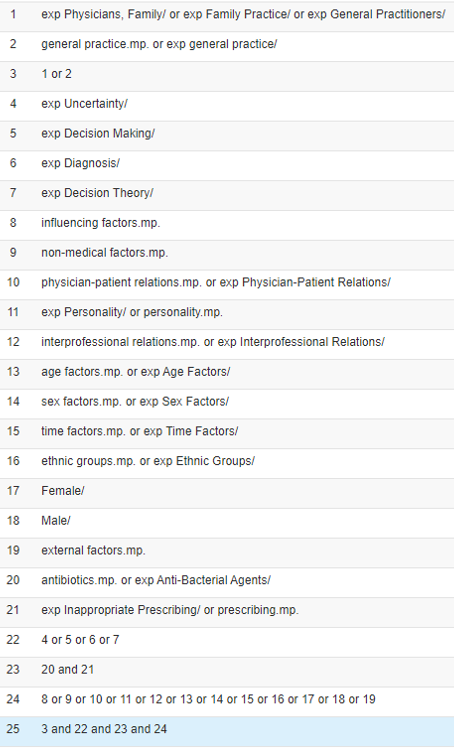


CINAHL:

Restricted to full text, academic journals only:

1. family physician
2. or family practice or family medicine or primary care
3. or general practitioner or gp or family doctor or primary care
4. and uncertainty
5. and decision making

update:

focusing on antibiotic prescribing:

family physician or family practitioner or general practice or general practitioner or primary care or primary health care or primary healthcare

and

decision making or decision-making or decision making process or decision-making process

and

antibiotic prescribing

from 1998-2019
