# Supplementary material for: Context and general practitioner decision-making - a scoping review of contextual influence on antibiotic prescribing
Source: BMC Fam Pract. 2021 Nov 15;22:225. doi: 10.1186/s12875-021-01574-x (PMC8591810; doi:10.1186/s12875-021-01574-x)
Supplement: Supplementary file 2 — Additional file 2. [file 12875_2021_1574_MOESM2_ESM.docx]

| Authors | Year | Title | Location | Methodology | Themes addressed | | | | | | |
| --- | --- | --- | --- | --- | --- | --- | --- | --- | --- | --- | --- |
|  |  |  |  |  | Space & place | Time, stress & emotion | Patient characteristics | Therapeutic relationship | Negotiating decisions & practice style | Managing uncertainty | Clinical experience |
| Aabenhus R, Siersam V, Sandholt H, Køster-Rasmussen R et al^31^ | 2017 | Identifying practice-related factors for high-volume prescribers of antibiotics in Danish general practice | Denmark | Quantitative | x | x |  |  |  |  |  |
| Akkerman AE, Kuyvenhoven MM, van der Wouden JC, Verheij TJ^63^ | 2005 | Determinants of antibiotic overprescribing in respiratory tract infections in general practice | Netherlands | Quantitative |  |  |  |  | x |  |  |

| Authors | Year | Title | Location | Methodology | Themes addressed | | | | | | |
| --- | --- | --- | --- | --- | --- | --- | --- | --- | --- | --- | --- |
|  |  |  |  |  | Space & place | Time, stress & emotion | Patient characteristics | Therapeutic relationship | Negotiating decisions & practice style | Managing uncertainty | Clinical experience |
| Ashdown HF, Räisänen U, Wang K, Ziebland S et al^52^ | 2016 | Prescribing antibiotics to ‘at-risk’ children with influenza-like illness in primary care: qualitative study | UK | Qualitative interviews |  | x |  | x |  |  |  |
| Björnsdóttir I, Hansen EH^46^ | 2002 | Ethical dilemmas in antibiotic prescribing: analysis of everyday practice | Iceland | Qualitative interviews and observation |  | x |  |  | x | x |  |

| Authors | Year | Title | Location | Methodology | Themes addressed | | | | | | |
| --- | --- | --- | --- | --- | --- | --- | --- | --- | --- | --- | --- |
|  |  |  |  |  | Space & place | Time, stress & emotion | Patient characteristics | Therapeutic relationship | Negotiating decisions & practice style | Managing uncertainty | Clinical experience |
| Björnsdóttir I and Holme Hansen E^39^ | 2002 | Intentions, strategies and uncertainty inherent in antibiotic prescribing | Iceland | Qualitative |  | x | x |  |  |  |  |
| Björnsdóttir, I., Kristinsson, K.G. & Hansen, E.H^72^ | 2010 | Diagnosing infections: a qualitative view on prescription decisions in general practice over time | Iceland | Qualitative – observations and interviews |  |  |  |  |  | x | x |

| Authors | Year | Title | Location | Methodology | Themes addressed | | | | | | |
| --- | --- | --- | --- | --- | --- | --- | --- | --- | --- | --- | --- |
|  |  |  |  |  | Space & place | Time, stress & emotion | Patient characteristics | Therapeutic relationship | Negotiating decisions & practice style | Managing uncertainty | Clinical experience |
| Bradely CP^51^ | 1992 | Factors which influence the decision whether or not to prescribe: the dilemma facing general practitioners | UK | Qualitative interviews |  | x |  |  | x |  | x |
| Butler CC, Rollnick S, Pill R, Maggs-Rapport F I *et al*^54^ | 1998 | Understanding the culture of prescribing: qualitative study of general practitioners' and patients' perceptions of antibiotics for sore throats | UK | Qualitative - interviews |  |  |  | x |  | x | x |

| Authors | Year | Title | Location | Methodology | Themes addressed | | | | | | |
| --- | --- | --- | --- | --- | --- | --- | --- | --- | --- | --- | --- |
|  |  |  |  |  | Space & place | Time, stress & emotion | Patient characteristics | Therapeutic relationship | Negotiating decisions & practice style | Managing uncertainty | Clinical experience |
| Buusman A, Andersen M, Merrild C, Elverdam B^68^ | 2007 | Factors influencing GPs' choice between drugs in a therapeutic drug group. A qualitative study. | Denmark | Qualitative interviews |  |  |  |  | x |  | x |
| Chauhan BF, Jeyaraman MM, Mann AS, Lys J *et al*^48^ | 2017 | Behavior change interventions and policies influencing primary healthcare professionals' practice-an overview of reviews. | Multinational | Overview of reviews |  | x |  |  |  |  |  |

| Authors | Year | Title | Location | Methodology | Themes addressed | | | | | | |
| --- | --- | --- | --- | --- | --- | --- | --- | --- | --- | --- | --- |
|  |  |  |  |  | Space & place | Time, stress & emotion | Patient characteristics | Therapeutic relationship | Negotiating decisions & practice style | Managing uncertainty | Clinical experience |
| Coenen S, Michiels B, Renard D, Denekens J, *et al*^59^ | 2006 | Antibiotic prescribing for acute cough: the effect of perceived patient demand | Belgium | Quantitative |  |  |  |  | x |  |  |
| Coenen, S., Michiels, B., Van Royen, P. Van der Auwera J *et al*^47^ | 2002 | Antibiotics for coughing in general practice: a questionnaire study to quantify and condense the reasons for prescribing | Belgium | Qualitative questionnaire |  | x |  |  | x |  | x |

| Authors | Year | Title | Location | Methodology | Themes addressed | | | | | | |
| --- | --- | --- | --- | --- | --- | --- | --- | --- | --- | --- | --- |
|  |  |  |  |  | Space & place | Time, stress & emotion | Patient characteristics | Therapeutic relationship | Negotiating decisions & practice style | Managing uncertainty | Clinical experience |
| Cordoba. G, Siersma. V, Lopez-Valcarcel. B, Bjerrum. L *et al*^29^ | 2015 | Prescribing style and variation in antibiotic prescriptions for sore throat: cross-sectional study across six countries | multinational | Quantitative | x |  | x |  | x |  |  |
| Denig P, Witteman CL, Schouten HW^65^ | 2002 | Scope and nature of prescribing decisions made by general practitioners | Netherlands | Qualitative – think aloud vignettes |  |  |  |  | x |  | x |

| Authors | Year | Title | Location | Methodology | Themes addressed | | | | | | |
| --- | --- | --- | --- | --- | --- | --- | --- | --- | --- | --- | --- |
|  |  |  |  |  | Space & place | Time, stress & emotion | Patient characteristics | Therapeutic relationship | Negotiating decisions & practice style | Managing uncertainty | Clinical experience |
| De Sutter AI, De Meyere MJ, De Maeseneer JM, Peersman WP^73^ | 2001 | Antibiotic prescribing in acute infections of the nose or sinuses: a matter of personal habit? | Belgium | Quantitative |  |  |  |  |  |  | x |
| Di Caccavo A, Reid F^40^ | 1995 | Decisional conflict in general practice: Strategies of patient management | UK | Qualitative interviews |  | x | x |  | x | x |  |

| Authors | Year | Title | Location | Methodology | Themes addressed | | | | | | |
| --- | --- | --- | --- | --- | --- | --- | --- | --- | --- | --- | --- |
|  |  |  |  |  | Space & place | Time, stress & emotion | Patient characteristics | Therapeutic relationship | Negotiating decisions & practice style | Managing uncertainty | Clinical experience |
| Drewniak D, Krones T, Sauer C, Wild V^41^ | 2016 | The influence of patients’ immigration background and residence permit status on treatment decisions in health care. Results of a factorial survey among general practitioners in Switzerland | Switzerland | Quantitative |  |  | x |  |  |  |  |
| Eggermont, D., Smit, M.A.M., Kwestroo, G.A. Verheij RA *et al*^38^ | 2018 | The influence of gender concordance between general practitioner and patient on antibiotic prescribing for sore throat symptoms: a retrospective study | Netherlands | Quantitative |  |  | x |  |  |  |  |

| Authors | Year | Title | Location | Methodology | Themes addressed | | | | | | |
| --- | --- | --- | --- | --- | --- | --- | --- | --- | --- | --- | --- |
|  |  |  |  |  | Space & place | Time, stress & emotion | Patient characteristics | Therapeutic relationship | Negotiating decisions & practice style | Managing uncertainty | Clinical experience |
| Duane S, Domegan c, Callan A, Galvin S *et al*^66^ | 2016 | Using qualitative insights to change  practice: exploring the culture of  antibiotic prescribing and consumption  for urinary tract infections | Ireland | Qualitative interviews |  |  |  |  | x |  |  |
| Garcia-Retamero R, Galesic M^71^ | 2014 | On defensive decision making: how doctors make decisions for their patients | Spain | Quantitative |  |  |  |  |  | x |  |

| Authors | Year | Title | Location | Methodology | Themes addressed | | | | | | |
| --- | --- | --- | --- | --- | --- | --- | --- | --- | --- | --- | --- |
|  |  |  |  |  | Space & place | Time, stress & emotion | Patient characteristics | Therapeutic relationship | Negotiating decisions & practice style | Managing uncertainty | Clinical experience |
| Geneau, R., Lehoux, P., Pineault, R, Lamarcge P^44^ | 2008 | Understanding the work of general practitioners: a social science perspective on the context of medical decision making in primary care | Canada | Qualitative interviews |  | x |  | x | x |  |  |
| Hajjaj FM, Salek MS, Basra MK, Finlay AY^60^ | 2010 | Non-clinical influences on clinical decision-making: a major challenge to evidence-based practice | UK | Literature Review |  |  |  |  | x |  |  |

| Authors | Year | Title | Location | Methodology | Themes addressed | | | | | | |
| --- | --- | --- | --- | --- | --- | --- | --- | --- | --- | --- | --- |
|  |  |  |  |  | Space & place | Time, stress & emotion | Patient characteristics | Therapeutic relationship | Negotiating decisions & practice style | Managing uncertainty | Clinical experience |
| Hardy-Holbrook R, Aristidi S, Chandnani V, Dewindt *et al*^62^ | 2013 | Antibiotic resistance and prescribing in Australia: current attitudes and practice of GPs | Australia | Quantitative |  |  |  |  | X |  |  |
| Horwood J, Cabral c, Hay AD, Ingram J^45^ | 2016 | Primary care clinician antibiotic prescribing decisions in consultations for children with RTIs: a qualitative interview study | UK | Qualitative interviews |  | x | x | x |  | x |  |

| Authors | Year | Title | Location | Methodology | Themes addressed | | | | | | |
| --- | --- | --- | --- | --- | --- | --- | --- | --- | --- | --- | --- |
|  |  |  |  |  | Space & place | Time, stress & emotion | Patient characteristics | Therapeutic relationship | Negotiating decisions & practice style | Managing uncertainty | Clinical experience |
| Jacoby A, Smith M, Eccles M^70^ | 2003 | A qualitative study to explore influences on general practitioners' decisions to prescribe new drugs | UK | Qualitative interviews |  |  |  |  | x |  | x |
| Jaye C, Tilyard M^67^ | 2002 | A qualitative comparative investigation of variation in general practitioners' prescribing patterns | New Zealand | Qualitative interviews |  |  |  |  | x | x | x |

| Authors | Year | Title | Location | Methodology | Themes addressed | | | | | | |
| --- | --- | --- | --- | --- | --- | --- | --- | --- | --- | --- | --- |
|  |  |  |  |  | Space & place | Time, stress & emotion | Patient characteristics | Therapeutic relationship | Negotiating decisions & practice style | Managing uncertainty | Clinical experience |
| Jaruseviciene, L., Radzeviciene-Jurgute, R., Lazarus, J.V, Jurgutis, A. *et al*^32^ | 2012 | A study of antibiotic prescribing: the experience of Lithuanian and Russian GPs | multinational | Qualitative focus groups | x |  |  | x | x |  |  |
| Kumar S, Little P, Britten N^36^ | 2003 | Why do general practitioners prescribe antibiotics for sore throat? Grounded theory interview study | UK | Qualitative interviews |  |  | x | x |  | x | x |

| Authors | Year | Title | Location | Methodology | Themes addressed | | | | | | |
| --- | --- | --- | --- | --- | --- | --- | --- | --- | --- | --- | --- |
|  |  |  |  |  | Space & place | Time, stress & emotion | Patient characteristics | Therapeutic relationship | Negotiating decisions & practice style | Managing uncertainty | Clinical experience |
| Loikas D, Karlsson L, von Euler M, Hallgren K^34^ | 2015 | Does patient's sex influence treatment in primary care? Experiences and expressed knowledge among physicians--a qualitative study | Sweden | Qualitative focus group |  |  | x |  |  |  |  |
| Macfarlane J, Holmes W, Macfarlane R, Britten N^57^ | 1997 | Influence of patients' expectations on antibiotic management of acute lower respiratory tract illness in general practice: questionnaire study | UK | Quantitative |  |  |  | x | x |  |  |

| Authors | Year | Title | Location | Methodology | Themes addressed | | | | | | |
| --- | --- | --- | --- | --- | --- | --- | --- | --- | --- | --- | --- |
|  |  |  |  |  | Space & place | Time, stress & emotion | Patient characteristics | Therapeutic relationship | Negotiating decisions & practice style | Managing uncertainty | Clinical experience |
| Mason A^69^ | 2008 | New medicines in primary care: a review of influences on general practitioner prescribing | UK | Literature review |  |  |  |  | x |  |  |
| Mazzaglia G, Caputi AP, Rossi A, Bettoncelli G *et al*^33^ | 2003 | Exploring patient- and doctor-related variables associated with  antibiotic prescribing for respiratory infections in primary care | Italy | Quantitative | x |  | x |  | x |  |  |

| Authors | Year | Title | Location | Methodology | Themes addressed | | | | | | |
| --- | --- | --- | --- | --- | --- | --- | --- | --- | --- | --- | --- |
|  |  |  |  |  | Space & place | Time, stress & emotion | Patient characteristics | Therapeutic relationship | Negotiating decisions & practice style | Managing uncertainty | Clinical experience |
| McIsaac WJ, Goel V^53^ | 1997 | Sore throat management practices of Canadian family physicians | Canada | Quantitative |  | x |  |  | x |  |  |
| McIsaac WJ, To T^58^ | 2004 | Antibiotics for lower respiratory tract infections. Still too frequently prescribed? | Canada | Quantitative |  |  |  |  | x |  |  |

| Authors | Year | Title | Location | Methodology | Themes addressed | | | | | | |
| --- | --- | --- | --- | --- | --- | --- | --- | --- | --- | --- | --- |
|  |  |  |  |  | Space & place | Time, stress & emotion | Patient characteristics | Therapeutic relationship | Negotiating decisions & practice style | Managing uncertainty | Clinical experience |
| Miller E, Mackeigan LD, Rosser W, Marshman J^64^ | 1999 | Effects of perceived patient demand on prescribing anti-infective drugs | Canada | Qualitative – interview and questionnaire of real consultations |  |  |  |  | x |  |  |
| Mousquès J, Renaud T, Scemama O^37^ | 2010 | Is the "practice style" hypothesis relevant for general practitioners? An analysis of antibiotics prescription for acute rhinopharyngitis | France | Quantitative |  | x | x |  |  | x |  |

| Authors | Year | Title | Location | Methodology | Themes addressed | | | | | | |
| --- | --- | --- | --- | --- | --- | --- | --- | --- | --- | --- | --- |
|  |  |  |  |  | Space & place | Time, stress & emotion | Patient characteristics | Therapeutic relationship | Negotiating decisions & practice style | Managing uncertainty | Clinical experience |
| Nazareth I, King M^55^ | 1993 | Decision making by general practitioners in diagnosis and management of lower urinary tract symptoms in women | UK | Quantitative |  |  |  | x | x |  |  |
| Petursson^27^ | 2005 | GPs' reasons for "non-pharmacological" prescribing of antibiotics. A phenomenological study | Iceland | Qualitative interviews | x | x |  | x | x | x |  |

| Authors | Year | Title | Location | Methodology | Themes addressed | | | | | | |
| --- | --- | --- | --- | --- | --- | --- | --- | --- | --- | --- | --- |
|  |  |  |  |  | Space & place | Time, stress & emotion | Patient characteristics | Therapeutic relationship | Negotiating decisions & practice style | Managing uncertainty | Clinical experience |
| Portnoy DB, Han PK, Ferrer RA, Klein WM, *et al*^43^ | 2013 | Physicians' attitudes about communicating and managing scientific uncertainty differ by perceived ambiguity aversion of their patients | USA | Quantitative |  |  | x |  |  |  |  |
| Schattner, A^35^ | 2014 | Are Physicians' Decisions Affected by Multiple Nonclinical Factors? | Israel | Qualitative interviews |  | x | x |  | x |  |  |

| Authors | Year | Title | Location | Methodology | Themes addressed | | | | | | |
| --- | --- | --- | --- | --- | --- | --- | --- | --- | --- | --- | --- |
|  |  |  |  |  | Space & place | Time, stress & emotion | Patient characteristics | Therapeutic relationship | Negotiating decisions & practice style | Managing uncertainty | Clinical experience |
| Scott A, Shiell A, King M^42^ | 1996 | Is general practitioner decision making associated with patient socio-economic status? | Australia | Quantitative |  |  | x |  |  |  |  |
| Sirota M, Round T, Samaranayaka S, Kostopoulou O^61^ | 2017 | Expectations for antibiotics increase their prescribing: Causal evidence about localized impact | UK | Quantitative |  |  |  |  | x |  |  |

| Authors | Year | Title | Location | Methodology | Themes addressed | | | | | | |
| --- | --- | --- | --- | --- | --- | --- | --- | --- | --- | --- | --- |
|  |  |  |  |  | Space & place | Time, stress & emotion | Patient characteristics | Therapeutic relationship | Negotiating decisions & practice style | Managing uncertainty | Clinical experience |
| Stocks NP, Fahey T^56^ | 2002 | The treatment of acute bronchitis by general practitioners in the UK. Results of a cross sectional postal survey. | UK | Quantitative |  |  |  | x | x | x |  |
| Strandberg EL, Brorsson A, Hagstam c, Troein M *et al*^30^ | 2013 | "I'm Dr Jekyll and Mr Hyde": are GPs' antibiotic prescribing patterns contextually dependent? A qualitative focus group study. | Sweden | Qualitative focus groups | x | x |  | x |  | x |  |

| Authors | Year | Title | Location | Methodology | Themes addressed | | | | | | |
| --- | --- | --- | --- | --- | --- | --- | --- | --- | --- | --- | --- |
|  |  |  |  |  | Space & place | Time, stress & emotion | Patient characteristics | Therapeutic relationship | Negotiating decisions & practice style | Managing uncertainty | Clinical experience |
| Tsiantou. V, Shea. S, Martinex. L, Agius. D *et al*^28^ | 2013 | Eliciting general practitioners' salient beliefs towards prescribing: A qualitative study based on the Theory of Planned Behaviour in Greece | Greece | Qualitative focus groups | x |  | x | x | x | x |  |
| Weiss MC, Fitzpatrick R, Scott DK, Goldacre MJ^49^ | 1996 | Pressures on the general practitioner and decisions to prescribe | UK | Quantitative |  | x |  |  | x |  |  |

| Authors | Year | Title | Location | Methodology | Themes addressed | | | | | | |
| --- | --- | --- | --- | --- | --- | --- | --- | --- | --- | --- | --- |
|  |  |  |  |  | Space & place | Time, stress & emotion | Patient characteristics | Therapeutic relationship | Negotiating decisions & practice style | Managing uncertainty | Clinical experience |
| Williams S J, Halls A V, Tonkin-Crine S, Moore M V *et al*^50^ | 2018 | General practitioner and nurse prescriber experiences of prescribing antibiotics for respiratory tract infections in UK primary care out-of-hours services (the UNITE study) | UK | Qualitative interviews |  | x |  |  |  | x |  |

*Table 2 – Details of Papers Included & Themes Contained Within*
